# An innovative DEI approach for emerging HIV investigators on Latino health

**DOI:** 10.1017/cts.2025.10218

**Published:** 2025-12-12

**Authors:** David M. Stoff

**Affiliations:** George Washington University, Department of Psychological and Brain Sciences, Washington, DC, USA

**Keywords:** Latinos, mentorship, lived experience, Diversity, Equity, and Inclusion (DEI), HIV

## Abstract

This commentary discusses research workforce programs designed to enhance the representation and engagement of Latinos in HIV research, highlighting key challenges and proposing actionable strategies for improvement. Mentorship education and cultural inclusivity are identified as the most salient issues because the former leads to stronger health-related outcomes and is linked to cognitive-and career-related factors while the latter offers the potential to directly dismantle structures of inequity. This commentary suggests recasting of Diversity, Equity, and Inclusion (DEI) initiatives from eligibility as Latino self-identification, to all individuals’ lived experiences and/or prior experience in service/research activities. Some issues and constructs (i.e., heterogeneity, perseverance, acculturation, cultural values), typically important for certain underrepresented minoritized groups in diversity workforce programs, are reinterpreted for their relevance to all potential participants. This commentary proposes a holistic approach to trainee eligibility, creating a more inclusive environment that respects both individuality and diversity, and, importantly, contributing to DEI does not require being a member of an underrepresented minoritized population group.

## Introduction

Hispanics/Latinos belong to the largest and fastest growing minoritized/marginalized subgroup in the United States, representing 13% of the total US population in 2000, accounting for nearly 20% in 2023, and projected to grow to 28% by 2060 [1]. As of 2022, Hispanics/Latinos constituted approximately 63.7 million or 19% of the US population, representing a 77% growth rate since 1980 (14.5 million) [2]. Despite their sizable and rapid growth, Hispanics/Latinos are significantly underrepresented at all levels of academic programs critical for entry to health profession careers. Currently, Hispanics/Latinos make up only a small portion of doctoral awardees in the sciences and public health, four percent of the public health faculty, six percent of physicians, and five percent of the NIH investigator pool [3], which is disproportionate to their population representation in the USA (19%) and health needs [1]. “Latino” refers to anyone born in or with ancestors from Latin America, including Brazilians, while “Hispanic” generally refers to people from Spanish-speaking Latin America including those countries/territories of the Caribbean or from Spain itself. For this article, Hispanics/Latinos are referred to as “Latinos,” since most “Hispanics” in the USA trace their ancestry to Latin American. Also, the term “Latinos,” rather than “Latinx” or Latine is used as an alternative gender-neutral label, because “Latinos” is more broadly preferred to describe the “Hispanic” or “Latino” population overall [4].

The underrepresentation of Latino researchers likely hinders research on Latino health and the ability to effectively address health inequities. The contemporary public health research community has embraced the call for social justice, diversity, inclusiveness, and health equity [5,6]. However, Latinos continue to be systematically excluded from faculty and leadership roles within academic public health schools and programs [7]. Public health training requires being trained by people from diverse backgrounds and backgrounds who embody and understand the need to dismantle the systems and structures that contribute to these inequities. For example, structural racism may operate in mental health organizations to undermine workforce diversity efforts and reinforce inequities [8]. Unless there is more consideration of structural racism (e.g., income inequities, biases, and discrimination) as a possible explanation in workforce diversity efforts, programs to increase diversity and inclusion and eliminate health disparities are likely to have limited effects [9]. Thus, it is crucial to rectify the lack of diversity in the research workforce to address the complex health problems that this population faces which are rooted in social, economic, and political systems that sustain inequity. Latinos represent a critical resource of talent that could be cultivated, if the above described constraints are overcome, in efforts to expand the research workforce. There is a relative dearth of HIV investigators from the Latino community, even though Latinos are disproportionately impacted by HIV and other STDs in the USA and are, thus, a historically minoritized/marginalized population at elevated risk for HIV infection. In 2020, Latinos had 3.6 times (per 100,000) higher HIV diagnosis and 2.1 times (per 100,000) higher mortality rates than White people in the USA [10]. In addition, the estimated number of new HIV infections among Latino males has increased in recent years [10]. Latino disparities are seen across the care continuum related to diagnosis, linkage to care and viral suppression [10]. Although our interest has been in expanding the HIV research workforce, these considerations below may apply to other fields and biomedical disciplines.

This commentary reviews current workforce strategies aimed at enhancing Latino representation in HIV research, examines common limitations, and proposes a more holistic, person-centered approach to address persistent gaps. Two of the most critical shortcomings of Latino research workforce programs (i.e., mentorship education and inclusive welcoming climate) are highlighted, and it is suggested that these obstacles may also apply to other investigators underrepresented in Biomedical or Social Science Research (BSSR) (i.e., African Americans, Latinos, Native Americans, and Pacific Islanders). This commentary expands upon a novel approach to eligibility for programs of Diversity, Equity, and Inclusion (DEI) (i.e., full, fair, and valued participation by all people) [11] and proposes the use of a more holistic approach to trainee program eligibility, including all individuals as potential participants, focusing on relevant lived experiences and unique professional/service backgrounds, rather than targeting the racial/ethnic population of Latinos (or any other specific underrepresented racial/ethnic population).

### Research workforce strategies for Latinos

The NIH and numerous academic institutions have made considerable investments to diversify the biomedical and behavioral research workforce by supporting a number of long-standing research initiatives and research training programs [12–14] designed to enhance the pool of BSSR investigators in the research workforce. Many of these programs have yielded outcomes that facilitated research development over a wide range of career stages among underrepresented BSSR investigators, including increases in: critical research skills [15]; confidence building via self-efficacy [16]; publication productivity [17,18]; health of historically minoritized/marginalized groups and health equity grant submissions [14]; and career/academic transitions [19]. These programs have been mainly for the aggregate of historically minoritized/marginalized groups with few tailored to the needs and experiences of a specific underrepresented group. While a useful first approach to develop research workforce strategies for all underrepresented BSSR investigators in general, this ignores the significant differences between and within historically minoritized/marginalized groups, especially for the Latino population [20]. It has been reported how a focus on the Latino population or other historically marginalized groups would strengthen trainee engagement and mentorship through tailoring and personalization of HIV programs [21]. Research workforce strategies, exclusively targeting Latinos, have utilized institutional-rich resources, such as Centers for AIDS Research [22–29], and most have achieved some measure of success in terms of improved academic, professional, and research outcomes [22,25–27,29]. Positive features of these programs include year-round training period, availability of junior and senior mentors, rich databases, and dedicated infrastructure that would support didactic, professional skills, grantsmanship, and research opportunities [22,26,29].

### Limitations of research workforce strategies for Latinos: fostering mentorship education and inclusive learning environment

**Figure 1.**
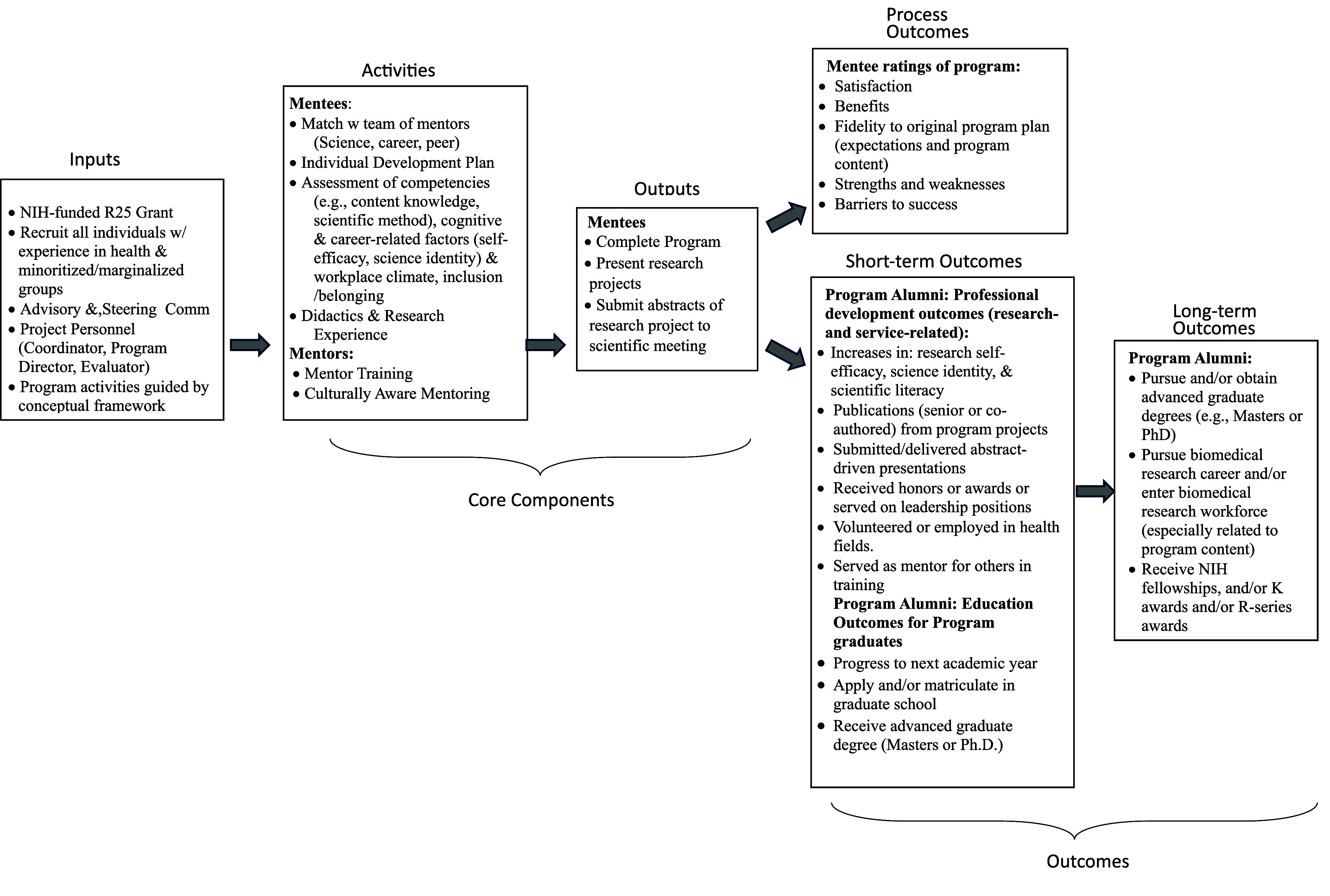
Logic model for DEI-related workforce programs.

A logic model, adapted from [30], as in Figure 1, was used to illustrate the core components involved in research workforce programs and guide their planning, implementation, and evaluation.

With this model as a roadmap, specific parts of diversity workforce programs that need attention can be identified. Programs need to foster an inclusive mentorship culture, particularly in view of multiple findings that mentorship availability contributes significantly to the diversity gap and dearth of scientists from underrepresented groups in the research workforce [31]. It is crucial not to lose sight that the success of any racial/ethnic diversity program is measured not only by head counts and publications but also by creating an equitable scientific community through a culture of inclusive mentorship. This would better ensure that mentees are not only equipped with methodological expertise to ultimately tackle public health challenges but also with a commitment to the central principles of DEI. This approach of inclusive mentorship has been proposed in the field of epidemiology [32]. In essence this means that, to be effective, mentors should be equipped with attributes such as mentor training [33], including competency in culturally aware mentoring [34], given that these trainings improve mentors’ ability to communicate with mentees, foster independence, and promote professional development by addressing equity and inclusion. Mentor effectiveness is also accomplished by creating standards in the use of certain evidence-based tools like individual development plans and assessment of mentor competencies [35]; regularly discussing mentoring challenges; and evaluating mentorship quality and mentee progress. Mentorship training is also key for mentees. For example, the *“Mentoring Up”* training curriculum empowers mentees to be active participants in the mentoring relationship for mentees [36] and has been found to be effective in advancing mentee skills and promoting strategies to be more proactive in getting their mentoring needs met [37]. Equity-focused or social justice mentoring approaches, specifically designed for historically minoritized/marginalized groups, are recommended. Intersectional mentoring [38], rooted in the principles of intersectionality [39], and liberatory race-conscious mentoring [40] or critical race-theory informed mentoring [41], is encouraged rather than just addressing individual needs. Both approaches are designed to address some structural and institutional factors that play into racial imbalance, and promote cultural humility, self-reflection about the impact of systemic oppression on opportunities and experiences of mentees. The intersectional approach provides a more contextualized understanding through examining the dynamic interplay between intersecting identities (e.g., being gay, Latino) and intersecting structures (healthcare, immigration policy, institutionalized homophobia) [42]. This in turn aids in revealing how systems of power and oppression intersect to shape population health and health inequities. When race-concordant mentorship, the preferred approach for the majority of mentees from groups of historically minoritized/marginalized [43], is not available, culturally responsive and anti-racism mentorship training and education [44] would be appropriate to capture the context and nuances of lived experiences of emerging non-underrepresented investigators. Evaluation of culturally responsive mentorship training should be conducted [33] to confirm that culturally responsive mentorship was indeed implemented.

Although closing the diversity gap is critical to ensure improved representation of all racial/ethnic groups within the research workforce, some have argued that evidence-based strategies and best practices to improve DEI in the biomedical research workforce remain poorly understood and underused [45]. Last year’s decision by the US Supreme Court to strike down the consideration of students’ racial status in college admissions [46] has emboldened some who oppose advancements of DEI. Some [47] have claimed that the progress of current DEI approaches leads to divisiveness and feelings of isolation, and experiences of reverse discrimination, perceptions of tokenism. The war on DEI has been compounded by the Presidential Executive Order to eliminate federal DEI programs altogether [48]. Since the most heated opposition to DEI stems from the exclusive targeting and selection of individuals from specific minoritized/marginalized groups, some criticism may be overcome by selecting individuals from all racial/ethnic groups, based on their relevant life experiences (further described below in IV), Additionally, it is believed that a more nuanced position would be to recast DEI so that it cultivates a sense of belonging and creates an inclusive climate. Strategies to create an inclusive welcoming climate could center around trainings that focus on implicit biases and racial microaggressions [49], ensuring that participants and staff members in the workforce feel safe, valued, and productive [50], and providing an integrated institutional approach to inclusive excellence and leadership [51].

In addition to addressing mentorship education and instilling an inclusive welcoming program climate, some other limitations of existing HIV workforce reports for Latinos, as described elsewhere [18], is recommended. For example, programs should be grounded in a conceptual model; include cultural competency training and a contextual focus; provide retention and follow-up data; and conduct formal process and content evaluation utilizing logic models.

### Lived experience regarding Latino health and/or Latino communities

A more holistic approach to trainee program eligibility, including all individuals as potential participants, is proposed, rather than targeting the population of Latinos (or any other specific minoritized/marginalized population), as in previous workforce programs [12–14]. Although this manuscript focuses on issues related to racial/ethnic identity, the holistic approach conceptualizes other multiple identities related to the Latino experience (e.g., nationality, socioeconomic status, gender identity, immigration history, and proficiency in Spanish language – spoken, read, and written). Eligibility criteria for workforce programs should thoughtfully take into account an applicant’s lived experiences and unique background in service/research activities as well as multiple intersecting identities, such as race/ethnicity, nationality, socioeconomic status, gender identity, and proficiency in Spanish language (spoken, read, and written). It is likely that when lived experiences, relevant to service/research activities in Latino communities and personal identity, are employed as eligibility criteria, the greater majority of potential participants will be individuals from a Latino background, without specifically targeting their racial/ethnic status.

Lived experience and relevant background regarding applicants’ service to minoritized communities can be assessed through qualitative methodology (along dimensions of interest, accomplishment, and plans that align with and are relevant to a particular program), through a narrative essay, addressing not only relevant service, professional or historical experiences but also individuals’ perspective and personal identities. The types of lived experiences that have frequently been encountered include volunteering/working with organizations that provide health or other supportive services (e.g., education) to Latino communities, providing services directly to Latinos or their families (e.g., translation or advocacy), and describing the personal impact of racism and on their commitment to work with and for Latino communities. While lived experience serves as the major factor for eligibility, it is considered as part of a more comprehensive participant selection method that includes multiple other factors, such as academic transcripts and recommendations. Further, a possible key aspect of participants’ lived experience is the feelings of connectedness or inclusion that is engendered to cope with and face the challenges of successfully navigating in the training environment. This may in turn enable participants to appreciate how much their mentors and others cared about them and their progress so that they felt heard, understood and valued.

### Re-interpreting diversity-related issues in context of lived experience

Some issues and constructs, typically important for certain underrepresented racial ethnic/groups in diversity workforce programs are discussed below, and re-interpreted with respect to lived experiences for their relevance to all potential participants: *heterogeneity, perseverance, acculturation, and cultural values*. (i) *Heterogeneity.* Latinos (and other racial/ethnic groups) are not a monolithic population [20] and, when they are self-identified as an aggregate, potential differences in diversity and variations within and across Latino subgroups are obscured. Applying lived experience to applicants who may come from many different backgrounds, potential participants are asked to reflect on the origins of their interest in science and how that experience provides a bridge for those who are in the early stages of their science identify development. This serves to capture the diversity of ways (i.e., individual heterogeneity) that first-hand involvement of exposure to particular events or conditions influences the meaning and interpretation that one attaches to these experiences as they relate to their career trajectories. With its multidimensional and ever-changing aspects, the utility of lived experience lies in its ability to reveal the unique stories and histories of each of us. (ii) *Perseverance*. Lived experience potentially gives more importance to characteristics of grit, determination, and perseverance. This originates from a model of student persistence, dependent on the extent of successful integration into the social and academic structures of the institution [52]. The focus on perseverance has more recently been updated in social cognitive career theory (SCCT) [53] which aids in the explanation of career choices and interests across racial/ethnic groups. SCCT has been n employed as a conceptual framework in diversity workforce training programs [54]. Self-efficacy (confidence in one’s ability to successfully perform a given tasks) is a core construct within SCCT [55]. Assessment of self-efficacy is suggested, along with science identity which is predicted by critical mentoring practices [41] to be a core attribute of effective mentoring relationships [55] and also predictive of student persistence and interest in science [56]. (iii) *Acculturation.* A key aspect of the experience of underrepresented BSSR investigators in the USA is acculturation, traditionally defined as adapting to a new environment and potentially adopting its values and practices. An acculturation gradient with variation among trainees is expected with diversity in characteristics such as acculturative stress, social support networks, or language proficiency. Acculturative stress may be a consequence of the cultural differences, and several reports have shown that social support may be a buffer against experienced stress [57]. A useful analogy for understanding the steps and practices of acculturation in the research process has been suggested by likening the research process to that of cultural integration [58], wherein emerging underrepresented BSSR investigators learn a new culture while retaining their own identity and membership in their minoritized/marginalized communities. This type of cultural integration into research and science may occur throughout the research training process from orientation to mentoring to skills development to research project implementation to manuscript and grant writing to securing funding. From this perspective, lived experience is conceptualized as acculturation-in-context wherein the support network of one in training is critical. The presence of a support network can greatly facilitate aspects of this professional socialization process and help to relieve some of the obstacles and stress inherent in navigating through the various steps of this process. (iv) *Cultural values.* By understanding and appreciating core Latino values [59], non-Latino individuals can engage more meaningfully with Latino communities, creating inclusive environments that recognize and honor their cultural strengths. Lived experiences and relevant first-hand involvement might include exposure to cultural values of the racial/ethnic population to be focused in either research or service components. Among the key cultural values that shape the socialization of Latino children is ***respeto*** (respect), which focuses on understanding the appropriate level of politeness and decorum for a particular situation, as well as defining the limits of acceptable behavior. Another essential value is ***educado*** (well-educated or well-mannered), which encompasses more than academic learning. It reflects the emphasis on strong socialization, including behaviors like self-control, fulfilling role responsibilities, obedience, respect, and self-sufficiency, all of which influence how Latino children interact in classroom settings. Additionally, ***familismo*** (familism), a cultural orientation that prioritizes collective needs over individual desires, highlights interacting and a deep sense of loyalty, reciprocity, and solidarity within both nuclear and extended family units, fostering strong family attachment and identification, providing the perspective and context for heightened religiousness and spirituality. These Latino cultural values favor the inclusion of Latinos of indigenous descent, while at the same time, they have become so ingrained into the culture of our nation and are relevant to all, so as to make the best use of the Latino cultural values. Thus, these are not unique values and competencies of specific groups but need to be acknowledged and used to the benefit of anyone engaged in research, training, and service. These cultural values may not be generalizable to all Latinos and mayo exist to varying degrees in non-Latinos with experience relevant to Latino health and Latino communities.

A challenge regarding the above issues, and perhaps to a greater degree for the core cultural values, is the insider vs outsider status of the researcher. Regarding the training relationship, the “insider” refers to the research mentor and the trainee holding the same status (e.g., Latino), while the “outsider” refers to holding a different status (e.g., non-Latino). Empirically, both “insider” and “outsider” research projects have advantages and limitations. While the “insider” may benefit from more rapport and ease of access with participants, they may create a potential power imbalance. Through their combination of lived experiences and relevant professional activities, research mentors bring a critical “insider” perspective to enriching an understanding of the Latino experience. While the “outsider” may be more objective, they share less of an intimate knowledge and act more in the role of an ally. Since there are major methodological and ethical issues that blur the insider-outsider distinction [60], it has been proposed that researchers may be both “insider” and “outsider” simultaneously, and these presumed extremes may lie on a continuum rather than a strict dichotomy [61]. This notion is consistent with our focus on lived experiences in all participants as it implies that characteristics of underrepresented groups and non-underrepresented groups are distributed in varying degrees for a given individual. The concept that the “insider”-“outsider” identities are interconnected and not static is described elsewhere [62]. A more nuanced perception of insider status as a continuum suggests the importance of building belonging with mentors (or the program). Conceptualizing belonging as a component of inclusion [63], it has been assessed through surveys on perceptions of workplace climate in a training environment [47]. Inclusion can be enhanced by community involvement, presence of reciprocity, shared language, and research familiarity/knowledge. Further, “insider-outsider” status may be related to high quality mentoring relationships through fostering an inclusive and safe learning environment [64,65]. A welcoming positive atmosphere encouraging a culture of mentorship is key to developing an environment of belonging, and this is likely to be more easily developed with “insiders” and race-concordant mentorship.

## Conclusion

In closing, this commentary maintains that the objectives of HIV research workforce strategies, (e.g., a DEI initiative to strengthen research on Latino health and for Latino communities), can be accomplished without the exclusive targeting of the Latino population, as in previously published reports. This can be done by embracing a more holistic person-centered approach to participant selection and thoughtfully consider the unique background and lived experiences of eligible mentees from all racial/ethnic groups. With a holistic approach, a more inclusive environment that respects both diversity and individuality can be created. Increased emphasis on psychosocial or sociocultural factors (e.g., implicit bias, stereotyped threat) is recommended to mitigate individual and institutional barriers to workforce diversity. This will in turn improve understanding of health equity and social justice and their impact on health, healthcare quality, and ultimately, health outcomes, as well as on the choices that all potential participants make along their career path. Further, such DEI initiatives will be fortified with a more comprehensive approach that includes increased attention to mentorship education, an inclusive welcoming climate, psychosocial variables that create and maintain equities, dismantling of structural-level barriers, improved acceptability, sustainability and retention, and, other relevant factors that impede diversification of the research workforce.
